# Early sexual debut: prevalence and risk factors among secondary school students in Ido-ekiti, Ekiti state, South-West Nigeria

**DOI:** 10.4314/ahs.v17i3.3

**Published:** 2017-09

**Authors:** Kabir Adekunle Durowade, Oluwole Adeyemi Babatunde, Lukman Omotayo Omokanye, Olusegun Elijah Elegbede, Lawrence Majekodunmi Ayodele, Kayode Razaq Adewoye1, Stella Adetokunbo, Charles O Olomofe, Adegboyega A Fawole, Oyebola Eyitayo Adebola, Temitope O Olaniyan

**Affiliations:** 1 Department of Community Medicine, Federal Medical Centre, Ido-Ekiti, Ekiti State, Nigeria; 2 Department of Obstetrics and Gynaecology, University of Ilorin, Ilorin, Nigeria; 3 Department of Behavioural Sciences, Federal Medical Centre, Ido-Ekiti, Ekiti State, Nigeria; 4 Department of Epidemiology and Community Health, University of Ilorin Teaching Hospital, Ilorin, Kwara State, Nigeria

**Keywords:** Sexual debut, prevalence, risk factors, Nigeria

## Abstract

**Background:**

Early adolescent sexual activity remains a recurring problem with negative psychosocial and health outcomes. The age at sexual debut varies from place to place and among different individuals and is associated with varying factors. The aim was to determine the prevalence and risk factors of early sexual debut among secondary school students in Ido-Ekiti, South-West Nigeria.

**Methodology:**

This was a cross-sectional study. The respondents were selected using multi-stage sampling technique. Pre-tested, semi-structured, self-administered questionnaire was used to collect data. Data was analyzed using SPSS version 15.

**Results:**

More than two-thirds, 40(67.8%), had early sexual debut. The prevalence of early sexual debut was about 11%. The mean age of sexual debut was 13.10±2.82; the mean age for early sexual debutants was 11.68±1.98. The mean number of sexual partners was 2.44±1.99. Male gender, having friends who engaged in sexual activities had association with early sexual exposure (p<0.05). Alcohol intake had the strongest strength of association for early sexual debut among the students.

**Conclusion:**

The high prevalence of early sexual exposure among the students calls for urgent interventions to stem the trend. This will help to reduce the devastating negative psycho-social and health sequels.

## Introduction

Early sexual debut (commonly defined as having had first sexual intercourse at or before age 14) and experience of sexual coercion or violence contribute to unintended adolescent pregnancy.^1^ Early age at sexual debut increases young people's risk of infection with HIV and other STIs.^2^ Besides being an important determinant of HIV infection, early age at sexual debut has negative effects on academic outcomes which can extend beyond secondary school, although concurrent changes in other psychosocial risk factors have not been investigated.^3^,^4^

Globally, early adolescent sexual activity remains a recurring public health issue.^5^ Early age at sexual debut is not without its accompanying complications^6^–^8^ which may range from an increased incidence of multiple sexual partners, unprotected sex, risk for sexually transmitted diseases including HIV/AIDs, unwanted and teenage pregnancies, unsafe abortions to mention a few.

Age at sexual debut varies from place to place and among different individuals, and is often due to varying factors. Among Nigerian adolescents aged 15–19 years, a fifth of them were found to have initiated sex (18% males and 22% females).^6^ Among Jamaican adolescents, the mean age at sexual debut was noted to be 11 years among girls and 15 among boys.^5^ In Ohio, USA, among adolescents aged 13–17 years, 8.6% of the adolescents aged 13 years admitted to having been sexually active before age 13. This number escalated with increasing age-17.7% before 14 years, 31.2% before 15years, 54.9% before 16 years and as high as 68.6% before 17 years.^7^

A number of factors have been identified as contributory to early age at sexual debut. For example mother's age at first sex has been found to be significantly associated with several of the children's early social behavior and their likelihood of being sexually active before the age of fourteen.^7^ Other factors that have been identified include substance and alcohol use, high prevalence of sexual initiation among peers, permissive norms about negative sexual outcomes, family economic disadvantage, large family size, minority group status, unstable family environment and low maternal education. Factors that have been identified as being protective on the other hand include religious inclination, older age of the adolescent, academic expectations, achievements, parental monitoring,^5^,^7^,^8^

This study therefore sought to identify the risk factors, determine the age at sexual debut and assess the prevalence and risk factors of early sexual debut among secondary school students in Ido-Ekiti, Ekiti State. For the purpose of our study, early sexual debut was taken as any penetrative sexual exposure at or before the age of 14 years irrespective of gender or circumstances.

## Methodology

Ido-Ekiti, a semi-urban community and headquarter of Ido/Osi Local Government Area (LGA) of Ekiti State, is located in the South-Western part of Nigeria. With a total land area of 332km^2^, the landmass enjoys friendly warm climate and fertile vegetation suitable for agricultural activities. Ido-Ekiti is bounded to the North by Usi-Ekiti; in the South by Igbole-Ekiti and Ora-Ekiti; in the East by Orin-Ekiti and Ipere-Ekiti and lastly to the West by Ilogbo-Ekiti. As at 2006, Ido-Osi LGA had a total population of 159,114 with vastly educated people. With an annual growth rate of 3.2%, the six year projected population will be 192,215.^9^–^11^

The people of Ido-Ekiti speak the Ekiti dialect of the Yoruba language. They are predominantly farmers growing both cash and food crops. These include Cocoa, Coffee, Kolanut, Yam, Maize and Okro among others. However, few of the people engage in vocational services. The people practice Christianity and Islam while some are traditional worshippers.

Certain noticeable social practices that are practiced among the people, especially the adolescents, include early penetrative sexual exposure, and associated multiple sexual partners with concomitant teenage pregnancy. Housing in certain segments of the community is over-crowded with concomitant poor sanitation and filthy environment, a reflection of the low socio-economic status of the people.

Ido-Ekiti had a total of six (three private and three public) secondary schools. These schools do not have guidance and counsellors and sex education was not in the curriculum of these secondary schools. Most of these schools are co-educational secondary schools.

The study was a descriptive/cross sectional study to identify the risk factors and determine the prevalence of early age at sexual debut among students of secondary schools in Ido-Ekiti, Ekiti State. The minimum sample size for the study was determined using Fisher's formula for estimating sample size to determine the prevalence or proportion of a factor where the population is greater than 10,000^12^. With 10% non-response rate, a total of 271 was obtained as sample size which was rounded up to 300. However, a final total of 365 respondents were sampled during the study.

A multistage sampling technique with four stages was used to select the respondents for the study. In the first stage, simple random sampling technique by balloting was used to pick four schools (two public and two private) out of the six secondary schools in the community. Thereafter, in the stage two, stratified sampling technique was used to divide the schools selected into strata based on class, i.e. JSS1 - SSS 3. In the stage three, simple random sampling technique by balloting was used to select two arms in each class. Weighted allocation was used to calculate the number of respondents that were selected from each of the selected arms of a class. In the final stage, simple random sampling by balloting was used to select the respondents that participated in each of the selected arms of a class.

A self administered, semi-structured questionnaire was used to elicit the study subjects' socio-demographic characteristics, age at sexual debut, factors responsible for early sexual debut and reproductive health parameters among the respondents. The questionnaire was pre-tested in another secondary school in Ilorin metropolis, Kwara State with a view of detecting deficiencies or ambiguities in the questionnaire and making appropriate corrections. Four research assistants participated and assisted in the data collection on the field. Data collation and editing was done manually to detect omission and ensure uniform coding. Data were analyzed using SPSS version 15 (IBM SPSS, Inc. Chicago, 2006); frequency tables and cross tabulations were generated to show the distribution across the socio-demographic variables and the presence of risk factors for early sexual debut. Bivariate analysis involving the use of Chi square, odds ratio with 95% confidence interval were employed to analyze the association among the variables. Chi-square test was used to determine statistical significance of observed differences in the cross tabulated variables. Odds ratio (OR) and Phico-efficient (φ) were calculated to determine the strength of association between the risk factors and early age at sexual debut. Ethical approval for the study was obtained from the research and ethics committee of the Federal Medical Centre, Ido-Ekiti. Informed assent/consent was obtained from the participants and the nature of the research was explained. Anonymity and confidentiality of the respondents' responses was ensured and guaranteed.

## Result

As shown in Table 1, the mean age of the respondents was 14.95±1.73. Approximately half of the students were males and more than three-quarters, 323(88.5%), were Christians. Also, as seen in Table 1, more than three-quarters of the respondents had their mothers and fathers alive. While about a third of them had fathers with tertiary education, about half of them had mothers with tertiary education.

**Table 1:** Socio-demographic and reproductive characteristics of the respondents (N=365)

Variable	Frequency(%)
**Age group (years)**	
<10	2 (0.6)
10–14	137 (37.5)
>14	226 (61.9)
Mean=14.95±1.73	
**Sex**	
Male	180 (49.3)
Female	185 (50.7)
**Religion**	
Christianity	323 (88.5)
Islam	42 (11.5)
**Mother Alive**	
Yes	352 (96.4)
No	13 (3.6)
**Father Alive**	
Yes	340 (93.2)
No	25 (6.8)
**Raised by who**	
Both parent	265 (72.6)
Father	24 (6.6)
Mother	51 (14.0)
Relative	25 (6.8)
**Father's Education**	
None	29 (7.9)
Primary	66 (18.1)
Secondary	151 (41.4)
Tertiary	119 (32.6)
**Mother's Education**	
None	31 (8.5)
Primary	82 (22.5)
Secondary	102 (27.9)
Tertiary	150 (41.1)
**Who do you live with**	
Both parent	236 (64.7)
Father	22 (6.0)
Mother	64 (17.5)
Relative	41 (11.2)
Others	2 (0.5)
**Relationship with parents**	
Very good	323 (88.5)
Good	30 (8.2)
Average	9 (2.5)
Poor	3 (0.8)
**Who do you have access to**	
Father	112 (30.7)
Mother	234(64.1)
Relative	16 (4.4)
Others	3 (0.8)
**Ever had a boy friend/girl** **friend**	
Yes	122 (33.4)
No	243 (66.6)
**Age at first menses(girls)** **(years) N=185**	
<10	0 (0.0)
10–14	167 (90.3)
>14	18 (9.7)
**Respondents' Class**	
Junior	55 (15.1)
Senior	310 (84.9)

Table 1 showed that about two-thirds, 236(64.7%), of them were living with both parents with more than three-quarters having good relationship with both parents. About two-thirds, 234(64.1%), of the respondents claimed they have good access to their mothers while just about a third, 112(30.7%), enjoyed good access to their fathers. A third of the respondents, 122(33.4%), ever had a boy/girl friend.

In Table 2, more than three quarters of them said their parents approved of, knew their friends and knew their movement in and out of the house. Similarly, more than three-quarters, 341(93.4%), were desirous of winning academic prize in the pursuit of their career. More than three-quarters, 321(87.9%), were not involved in any form of religious activities.

**Table 2:** Socio-demographic and reproductive characteristics of the respondents… cont'd

Variable	Frequency (%)
**Discuss pubertal/maturity** **issues with anyone**	
Yes	137 (37.5)
No	228 (62.5)
**If yes, with who**	
Mother	48 (13.2)
Father	7 (1.9)
Sibling	20 (5.5)
Friend	57 (15.6)
Relative	5 (1.4)
**Discuss effect of early sex** **with anyone**	
Yes	102 (27.9)
No	263 (72.1)
**If yes with whom n=102**	
Parent	19 (18.6)
Friend	67 (65.7)
Sibling	14 (13.7)
Teacher	2 (2.0)
**Discuss effect of Teenage** **pregnancy with anyone**	
Yes	94 (25.8)
No	271 (74.2)
**If yes, with whom n=94**	
Mother	10 (10.7)
Friend	69 (73.4)
Sibling	13 (13.8)
Teacher	2 (2.1)
**Rule at home guiding time** **of return and leaving**	
Yes	229 (81.9)
No	66 (18.1)
**Parent approve your** **friends**	
Yes	316 (87.4)
No	49(12.6)
**Parent know where you** **are during free time**	
Yes	306 (83.8)
No	59 (16.2)
**Like to win prize for** **academic excellence**	
Yes	341 (93.4)
No	24 (6.6)
**Won prize or recognition** **For academic excellence**	
Yes	276 (75.6)
No	89 (24.4)
**Involvement in religious** **activities**	
Not at all	341 (93.4)
Slightly	11 (3.0)
Really involved	13 (3.6)
**Mother/Father know most** **of your friends**	
Yes	334(91.5)
No	31(8.5)

Table 3 shows sexual exposure among the respondents. Less than one-quarter, 59(16.2%), ever had sex with anyone and out of this, more than two-thirds, 40(67.8%), had early sexual debut. However, the overall prevalence of early sexual debut among the respondents was about 11%. The mean age of sexual debut among the respondents was 13.10±2.82, while the mean age for early sexual debutants was 11.68±1.98. The mean number of sexual partners among the respondents was found to be 2.44±1.99 and about two-thirds, 38(64.4%), of them had between 1–2 sexual partners.

**Table 3:** Sexual exposure among the respondents

Variable	Frequency (%)	N=365
**Ever had sex with anyone before**		
Yes	59 (16.2)	
No	306 (83.8)	
**Age at sexual debut (years) n=59**		
<10	6(10.2)	
10–14	34(57.6)	
>14	19 (32.2)	
Mean= 13.12±2.80		
**Early sexual debut (years) n=40**		
<10	6 (15.0)	
10–14	34 (85.0)	
Mean= 11.68±1.98		
**Number of sexual partners n=59**		
1–2	38 (64.4)	
3–5	14 (3.8)	
≥6	7 (1.9)	
Mean=2.44±1.99		
**First person to have sex with n=59**		
Friend	56 (94.9)	
Husband	1 (1.7)	
Teacher	2 (3.4)	
**Circumstances surrounding first** **sexual exposure n=59**		
Willingly	28 (47.5)	
Unwillingly	14 (23.7)	
Influence of friends	12 (20.3)	
Influence of alcohol	3 (5.1)	
Others	2 (3.4)	
**Have most of your friends had sex before**		
Yes	91(24.9)	
No	274 (75.1)	

Also, in Table 3, more than three-quarters, 56(94.9%), of those with sexual exposure had it with either their boy or girl friend. Again, while 2(3.4%) of them claimed they had their first sexual experience with their school teachers, only a respondent said she had her first sex with her husband at the age of 15 years. On the circumstances surrounding first sexual exposure among the early sexual debutants, about half 28(47.5%), of them said it was willingly, less than a quarter, 14(23.7%) said it was unwillingly. Although, very few of them, 3(5.1%), said it was under the influence of alcohol; almost a quarter, 12(20.3%), said it was due to the influence of friends. In the same vein, a quarter, 91(24.9%), of the respondents had friends who engaged in sex.

In Table 4, the mean age of early sexual debut for males (11.34±2.058) was lower than that of the females (12.55±1.508), but the observed difference in means was not statistically significant (p=0.087). However, within the gender, the mean age of early sexual debut was generally lower than the mean chronological age of either the males or females. This observed difference was statistically significant for males (p=0.000) and females (p=0.006). As shown in Table 5, gender of the respondents was found to have association with early sexual debut as more males than females were found to have had early sexual debut. This observed difference between male and female sexes was statistically significant (OR=3.04, 95%CI=1.40–6.72, p=0.001, φ=0.16). Also, having friends who engage in sex and intake of alcohol were also significantly associated with early sexual exposure. About a quarter, 22(24.2%), of the respondents who had friends who engaged in sexual activities also had early sexual debut compared with less than one-tenth, 18(6.6%), among those whose friends did not engage in sex. This observed difference was found to be statistically significant (OR=4.53, 95%CI=2.19–9.42, p=0.0000032, φ=0.24). Also, more than two-thirds, 10(66.7%), of those who took alcohol had early sexual exposure compared with more than three-quarters, 320(91.4%), of those who did not take alcohol with no early sexual exposure. This observed difference was also found to be significant (OR=21.33, 95%CI=6.17–77.59, p=0.0000002, φ=0.37).

**Table 4:** Comparison of means of selected parameters among the respondents

Variables	Mean± SD	n	t-test	p value
**Age**				
Male	15.25±1.740	180		
Female	14.66±1.693	185	10.801	0.001
**Age of Sexual debut**				
Male	13.21±3.036	47		
Female	12.75±1.603	12	0.258	0.613
**Age of early sexual debut**				
Male	11.34±2.058	29		
Female	12.55±1.508	11	3.092	0.087
**Female**				
Age at menses	12.92±0.793	12		
Age at sexual debut	12.75±1.603	12	0.352	0.732
**Female**				
Age at menses	12.82±1.16	11		
Age at early sexual debut	12.55±1.508	11	0.539	0.602
**Male**				
Age	15.03±1.973	29		
Age at early sexual debut	11.34±2.058	29	7.022	0.000
**Female**				
Age	14.82±1.250	11		
Age at early sexual debut	12.55±1.508	11	3.508	0.006
**Male**				
Age	15.40±2.050	47		
Age at sexual debut	13.21±3.036	47	4.317	0.000
**Female**				
Age	14.92±1.240	12	3.606	0.004
Age at sexual debut	12.75±1.603	12		

**Table 5:** Factors associated with early sexual debut among the respondents

Variable	Early sexual exposure		χ^2^	OR*	95%CI	p value	φ
	Yes (%)	No (%)					
**Age (years)**							
<10	0(0.0)	2(100.0)	0.25	0.00	0.00–33.99	1.000*	0.03
≥10	40(11.0)	323(89.0)					
**Sex**							
Male	29(16.1)	151(83.9)	9.66	3.04	1.40–6.72	0.001	0.16
Female	11(5.9)	174(94.1)					
**Class in school**							
Junior	5(9.1)	50(90.9)	0.23	0.79	0.26–2.23	0.630	0.03
Senior	35(11.3)	275(88.7)					
**Leaving with**							
Parent	35(11.2)	277(88.8)	0.01	0.96	0.33–2.98	1.000*	0.005
Relative	5(11.6)	38(88.4)					
**Mother Alive**							
Yes	40(11.4)	312(88.6)	1.66	0.00	-	0.375*	0.07
No	0(0.0)	13(100.0)					
**Father Alive**							
Yes	39(11.5)	301(88.5)	1.33	3.11	0.43–63.42	0.501*	0.06
No	1(4.0)	24(96.0)					
**Friends engage in sex**							
Yes	22(24.2)	69(75.8)	21.70	4.53	2.19–9.42	0.0000032	0.24
No	18(6.6)	256(93.4)					
**Alcohol intake**							
Yes	10(66.7)	5(33.3)	49.75	21.33	6.17–77.59	0.0000002*	0.37
No	30(8.6)	320(91.4)					
**Involvement in religious** **activities**							
Yes	8(18.2)	36(81.8)	2.67	2.01	0.78–4.99	0.120*	0.09
No	32(10.0)	289(90.0)					
**Desire to win** **academic** **prize**							
Yes	36(10.6)	305(89.4)	0.86	0.59	0.18–2.17	0.318*	0.05
No	4(16.7)	20(83.3)					

OR= Odds Ratio

* Fishers exact χ=Phi co-efficient

Being in a junior or senior class also had no association with early sexual debut as about a tenth of respondents in each of junior or senior class had engaged in early sex and so the observed difference was not statistically significant (OR=0.79, 95%CI=0.26–2.23, p= 0.630). Also, respondents' involvement in religious activities had no association with early sexual exposure as more than three-quarters, 289(90.0%), of those who did not partake in religious activities had no early sexual debut. However, only about a fifth, 8(18.2%), of those involved in religious activities had early sex. This difference was not statistically significant (OR=2.67, 95%CI=0.78–4.99, p=0.120)

## Discussion

The mean age of respondents in this study was 14.95±1.73. This was not surprising as the mean age fell within the recognized school age of 5–15 years and besides our study population was secondary school students. However, this was lower than the mean age obtained in a similar study in Ethiopia with a mean age of 19.4±2.7.^13^ Though, in the two studies, more than half of the respondents were in the age group 15–19 years, the difference in the mean age could be due to the fact that the Ethiopian study was among youths while this study focused on secondary school students. Besides, the Ethiopian study was a comparative cross-sectional study of rural and urban areas while this study is simply cross-sectional.

Less than one quarter, 16.2%, of the respondents in this study had sexual exposure with more than two-thirds, 67.8%, of them having early sexual debut. This showed than more than three-quarters, 83.8%, of the respondents had no sexual experience. This was lower than the value obtained among youths in Ethiopia where half of them have ever had sex in both rural and urban communities' studied.^11^ The difference between the Ethiopia study and this study could be due to the fact that this study was among in-school adolescents while the one in Ethiopia was conducted among youth. It is also lower than the 39% obtained in a study among South African youths by Khaagelani et al where early sexual debut was pegged at 16 years.^14^ Similarly, it was found to be lower than the 48.7% reported to be sexually active in a study in Tanzania by Mmbaga et al.^4^ Though the Tanzania study and this study were conducted among in-school adolescents in secondary school, however, the proportion of males sampled were much higher in the Tanzania study than this study. While males made up 168(53.2%) in the Tanzania study, they constituted 49.3% in this study; and males tend to be more involved in early sexual debut than the females.

However, the findings in this study were similar to those of Fatusi et al in a study among Nigeria adolescents where more than three-quarters, 80.2%, of them reported having never had sex.^6^ This could be due to the fact that the Nigerian society/culture views early sexual exposure among adolescents as being morally wrong. Besides, some of the students may not want to disclose this especially the females.

On the circumstances surrounding the first sexual exposure, about half, 47.5%, said they willingly had the experience, while almost quarter, 23.7%, said it was not willingly done. This was lower than the value obtained in a study by Baumgartner et al in Jamaica where about one-third said they were forced to participate in their first sexual experience.^1^ The reason for the difference could be that the Jamaican study was a matched case-control between currently pregnant and sexually experienced but never pregnant controls.

Though the mean age of sexual debut among the respondents was 13.10±2.82, the mean age of the early sexual debutants was 11.68±1.98. This study also showed that the mean age of sexual debut was lower among females (12.75±1.603) than their male counterparts (13.21±3.036). However, the mean age of early sexual debut was lower among males (11.34±2.058) than females (12.55±1.508). However, the observed difference was not statistically significant (p>0.05). This was not at variance with the findings obtained by Mmbaga et al in Tanzania where the mean age at sexual debut for males (14.7±2.5 years) did not differ from that of females (14.6±2.0 years) with a p value of 0.416.4 However, these values were lower than the mean age of sexual debut obtained in the study in Ethiopia which obtained 16.8±2.55. Again, the study in Ethiopia was a comparative study of rural and urban areas while this study and that in Tanzania were cross-sectional studies conducted among secondary school students in a semi-urban/municipal area.

This study found that respondents' sex/gender was associated with early sexual exposure as more males than females had their first sexual experience at or before the age of 14 years. This observed difference was statistically significant with a p value of 0.001. This was not unexpected as males are more adventurous and desirous of trying new experiences as compared to the female gender. Also, males are more likely to report their first sexual exposure than females. This was buttressed in another study in Tanzania where 82.0% of males reported their sexual debut status as against 33.0% among the females.^15^

Also, having friends who engaged in sex and the intake of alcohol were also found to have association with early sexual exposure with p<0.05. This is so because these are social vices that can influence an adolescent towards bad behavior. Intake of alcohol, especially if excessive, can cause disorientation and loss of self-control. An adolescent, regardless of the sex difference, moving with friends who engage in sexual activities will also be negatively influenced towards engaging in pre-marital sex. A similar finding was obtained in Tanzania by Mmbaga et al where alcohol intake and having friends who engaged in sex had significant association with sexual debut.^4^ Similarly, in a study in Ethiopia by Mazengia et al, those who drink alcohol were two times (adjusted OR=2.16, 1.12–4.18) more likely to initiate sexual intercourse before the age of 18.^13^ However, the study pegged early age of sexual initiation as being < 18years compared with ≤14 years in this study. The strength of association, also established using the phi coefficient, showed that alcohol intake has the highest value compared with the other factors.Indeed, of all the significant risk factors for early sexual exposure, the intake of alcohol was also found to have the highest phi co-efficient (φ= 0.37) which implies the strongest strength of association for early sexual debut among the students.

## Conclusion/recommendation

Male gender, having friends who engaged in sex and the intake of alcohol were found to be risk factors for early sexual exposure among the students. The presence of these risk factors and the high prevalence of early sexual exposure among the students is a worrisome trend. Exposure to risk factors for early sexual exposure needs to be curtailed. Sale of alcohol to adolescents and alcohol sales outlet proximal to school environments need legislative restriction; sex education should be incorporated into curriculum of secondary schools. Parents have a moral duty to sex-educate their children on dangers of early sexual debut; and to censor the kind of friends their wards keep to avert negative social influence on them. Lastly, there is a dire need for urgent multiple intra-sectoral and inter-sectoral interventions to stem the trend and reduce the devastating negative psycho-social and health sequels of adolescents' early sexual debut.
